# Supplementary material for: Single-nucleus transcriptomics reveals functional compartmentalization in syncytial skeletal muscle cells
Source: Nat Commun. 2020 Dec 11;11:6375. doi: 10.1038/s41467-020-20064-9 (PMC7732842; doi:10.1038/s41467-020-20064-9)
Supplement: Supplementary file 10 — Description of Additional Supplementary Files [file 41467_2020_20064_MOESM10_ESM.docx]

**Legends for Supplementary Data files**

**Title:** Supplementary Data 1.

**Description:** Top 50 genes that distinguish each time point during regeneration.

**Title:** Supplementary Data 2.

**Description:** Numbers and frequencies of nuclei belonging to each cluster identified in this study.

**Title:** Supplementary Data 3.

**Description:** Full list of the marker genes for each cluster identified in this study.

**Title:** Supplementary Data 4.

**Description:** Top 10 transcription factors enriched in each cluster.

**Title:** Supplementary Data 5.

**Description:** Summary of sequencing samples, mouse conditions and sequencing parameters.

**Title:** Supplementary Data 6.

**Description:** List of primers used in this study for RT-qPCR and FISH experiments.
